# Supplementary material for: Factors associated with antenatal exercise in Arba Minch town, Southern Ethiopia: A community-based cross-sectional study
Source: PLoS One. 2022 Feb 22;17(2):e0260840. doi: 10.1371/journal.pone.0260840 (PMC8863279; doi:10.1371/journal.pone.0260840)
Supplement: S1 File — (DOCX) [file pone.0260840.s001.docx]

## Annex 1: CONSENT FORM

## Arba Minch University College of Medicine and Health Sciences Consent form for a research project entitled factors associated with antenatal exercise in Arba Minch town, Southern Ethiopia

**Hello**!

My name is _______________. I am working for investigator from Arba Minch University who are doing a research on factors associated with antenatal exercise in Arba Minch town, Southern Ethiopia. You have identified as a study participant hoping that you would be willing to help me by providing some information. I would like to ask you a few questions about your socio-demographic characteristics , obstetrical history and about antenatal exercise which may take 25-30 minutes.

The goal of this study is to assess the factors associated with antenatal exercise in Arba Minch town, Southern Ethiopia. All information you provide will be kept confidential. I will not include any identifiers, such as your name or exact address. Your role in the success of the research is important and I appreciate your contribution to the research.

You have a full right to refuse part or the whole questionnaires & no one enforces you to do so. However, your honest participation and answers to the questionnaire will help us in a better understanding of the problem and give guidance on how to intervene in the study area. So are you willing to participate actively and honestly?

I understood about the advantage of the research, the roles I will have in the research and have agreed to participate in the research. (*If yes, let her sign and go ahead, if No stop here*.)

Yes No

Signature of the data collector __________________________

Date: ______________________________________________

Annex -2: Assent form

My name is -------------------------I am asking you to take part in research study because of we are trying to learn more practice of antenatal exercise and associated factors among pregnant women in Arba Minch town Southern, Ethiopia. If you agree to be in this study since your child or sisters age is less than 18 years old and this age is not allowed in Ethiopia constitution to give information required. So you are kindly requested to give information from your child’s point of view or you are expected to more understand about the research and final agree that she can give information .I need you to provide me your honest answer to the questions you want to respond as this would help us to come up with genuine conclusions and recommendations that would be used as evidence. Participating in this research project will not have direct benefit to you also there is no risk that comes to as pregnant women. So, make sure that, there should be no harm caused because you are involved in this study. You have right not to start or terminate it if something unclear happen.

. I have understood what will be you doing for this study

All my questions have had answered

I have talked to your parents and agreed to take part in this research.

Do you agree to give the required information? Yes No

Or parents agree Yes No

Contact persons:

1. Maechel Maile Email:maechelmaile11@gmail.com phone: +251987174062

2. Mekdes Kondale Email: [kondale@gmail.com](mailto:kondale@gmail.com). Phone: 09 26 16 0903

3. Gebresilasea Gendisha Email: [gebretecno@gmail.com](mailto:gebretecno@gmail.com) Phone: 0934596503

Annex-3: Questions in English version

Part 1: questions related socio demographic characteristics

| Serial number | Characteristic’s | Alternative responses | Skip |
| --- | --- | --- | --- |
| Q.101 | What is your age? | Age in years----- |  |
| Q.102 | what is your religion | Orthodox-----------1 protestant----------2 Catholic------------3 Muslim ------------4  Others specify______99 |  |
| Q.103 | what is your current marital status | Single--------------1 married------------2  Divorced-----------3  Widowed-----------4 |  |
| Q.104 | What is your educational level? | Illiterate ----------1  Reading and writing---2  Elementary--------3 High School --------4  College or university---5 |  |
| 105 | What is educational level  Of your partner? | Illiterate ----------1  Reading and writing---2  Elementary--------3 High School --------4  College or university---5 |  |
| Q.106 | What is occupational background? | Employed in gov.t  sectors------------1  House wife--------------2  Private business trader-3  Employed in NGO----4 |  |
| Q.107 | What is your income level per a month? | ----------------Ethiopian birr |  |

Part: 2 Obstetrical histories

| Serial no | Question | Alternative |  |
| --- | --- | --- | --- |
| 201 | How many times in total you went for ANC visit in this pregnancy |  |  |
| Q.202 | How many times in total you become pregnant | One times-----1  2-4-------------2  Above 4--------3 |  |
| Q.203 | How many times in total you gave birth | none-----1  1--------------2  ≥2--------3 |  |
| Q.204 | How many children do you have? | No child------------1  1-2 children---------2  >2children and above--3 |  |
| Q.205 | Have you ever had history of miscarriage? | Yes---------1  No----------2 |  |
| Q.206 | How many months pregnant are you? | <4 months  4-6 months  7-9 months |  |
| Q.207 | How many times you attended antenatal care? | 1-2------------------1  3-4------------------2  More than 4 -------3 |  |

Part: 3 Question related to awareness of different type of antenatal exercise

| Serial no | Question | Alternative |  |
| --- | --- | --- | --- |
| Q.301 | Have you ever done physical exercise before becoming pregnant? | Yes-----------1  No------------2 |  |
| 302 | Have you ever heard about antenatal exercise? | Yes-----------1  No------------2 |  |
| Q.303 | If your answer is **YES,302** from where you heard? | mass media Tv…….1  health care provider at health institution…..2  Health extension workers health education-------3  Social media………4 |  |
| Q.304 | What types of antenatal exercises you heard or aware from the following?  (Possible more than one answer). ? | Walking -------------1 Aerobics ----------------2  Relaxation/Breathing-3  Pelvic Floor Exercises-4  Back Care Exercises--5  Ankle and toe exercise-6 |  |

Part four: Questions concerning knowledge antenatal exercise.

| Serial no | Question | Alternative |  |
| --- | --- | --- | --- |
| Q.401 | Do you thing ANE exercise during pregnancy reduces risk of back pain? | Yes--------------------1  No---------------------0 |  |
| Q.402 | Do you thing ANE Prevents excessive weight gain during pregnancy | Yes--------------------1  No---------------------0 |  |
| Q.403 | Do you thing exercise increased energy and stamina during pregnancy | Yes--------------------1  No---------------------0 |  |
| Q.404 | Do you thing ANE can help cope with labor and delivery pain | Yes--------------------1  No---------------------0 |  |
| Q.405 | Do you thing exercise during pregnancy can reduces risk of DM | Yes--------------------1  No---------------------0 |  |
| Q.406 | Do you thing ANE can decrease high blood pressure during pregnancy | Yes--------------------1  No---------------------0 |  |
| Q.407 | Do you thing ANE exercise helps more rapid postnatal recovery | Yes--------------------1  No---------------------0 |  |
| Q.408 | Do you thing ANE prevents antenatal and postnatal depression | Yes--------------------1  No---------------------0 |  |
| Q.409 | Do you thing ANE benefits general health and development of the baby | Yes--------------------1  No---------------------0 |  |
| Q.410 | Do you thing vaginal bleeding during pregnancy it is contraindicated to do ANE | Yes--------------------1  No---------------------0 |  |
| Q.411 | Do you thing uterine contractions is one of the contraindication to do ANE. | Yes--------------------1  No---------------------0 |  |
| Q.412 | Do you thing chest pain during pregnancy is contraindicated to do ANE | Yes--------------------1  No---------------------0 |  |
| Q.413 | Do you thing difficulty of breathing during pregnancy is contraindication to do ANE | Yes--------------------1  No---------------------0 |  |
| Q.414 | Do you thing Premature labour during pregnancy is contraindicated to do ANE | Yes--------------------1  No---------------------0 |  |
| Q.415 | Do you thing Poorly controlled type 1 Diabetic during pregnancy is contraindicated to do ANE | Yes--------------------1  No---------------------0 |  |
| Q.416 | Do you thing dizziness during pregnancy is contraindicated to do ANE | Yes--------------------1  No---------------------0 |  |

Part five: Question concerning attitude of towards antenatal exercise during pregnancy

| Serial no | Question | Strongly agree | Agree | uncertain | Disagree | Strongly disagree |
| --- | --- | --- | --- | --- | --- | --- |
| Q.501 | Do you feel Performing ANE exercise during pregnancy is necessary? | 4 | 3 | 2 | 1 | 0 |
| Q.502 | Do you feel Performing ANE exercise during pregnancy is risky to the fetus? | 4 | 3 | 2 | 1 | 0 |
| Q.503 | Do you feel antenatal exercise suit with our culture | 4 | 3 | 2 | 1 | 0 |
| Q.504 | Do you feel pregnant women should perform exercise under the guidance of health care professional | 4 | 3 | 2 | 1 | 0 |
| Q.505 | Do you feel Performing antenatal exercise can reduce pregnancy-related complications | 4 | 3 | 2 | 1 | 0 |
| Q.506 | Do you feel Practicing ANE exercise during pregnancy helps in post-delivery recovery | 4 | 3 | 2 | 1 | 0 |
| Q.507 | Do you feel ANE exercising will helps you get back to your shape | 4 | 3 | 2 | 1 | 0 |
| Q.508 | Do you feel antenatal exercise regimen should vary from One pregnant woman to another | 4 | 3 | 2 | 1 | 0 |

Part six: Question concerning practice of exercise during pregnancy

| Serial no | Question | Alternative |  |
| --- | --- | --- | --- |
| Q.601 | Do you practice any antenatal exercise in this current pregnancy? | Yes------------------1  No---------------------0 | If **no** skip to Q506 |
| Q.602 | What type of exercise you exercised now? (it is Possible more than one answer  ? | Walking ------------1  Aerobics------------2  Relaxation and breathing exercise-3  Pelvic floor exercise -----4  Back exercise----------5  Ankle and toe raising —6  Swimming------------7  Cycling---------------8 |  |
| Q.603 | Who advised (guided) you to do antenatal exercise during the present pregnancy? | Healthcare provider---1  Self- --------------------2  Other person -------3 |  |
| Q.504 | How many times per week you exercised | ≤Two times/week --1  ≥ three times/week-2 |  |
| Q.605 | For how many minute you exercised per session? | <30 mint -----------1  ≥30mnt--------------2 |  |
| Q.606 | Why you don’t exercise currently during this pregnancy? (you can circle more than) | 1.I don’t have information  2.My health professional hasn’t advised me to do exercise  3.Exercise will harm the baby  4. Lack of time  5.Exercise will harm me  6. I feel tired when I exercise  7.I am not in good health  8.Because of cultural reason  9.other specify--------------- |  |

Thank you very much for your cooperation!

Annex-5: Amharic translated questions

የስምምነት ማስገንዘቢያ ቅጽ

አርባምንጭ ዩኒቨርሲቲ የጤና ሳይንስ ኮሌጅ ሚዴዋይፈሪ ትምህርት ክፍል

እንደምን አደሩ/አረፈዱ፡፡ስሜ ------------------- ሲሆን የምሰራው በ---------------- ነው፡፡

ነፍሰጡር ሴቶች ስለቅድመወልድ የአካል እንቅስቃሴ መስራትናተያያዥ ምክንያቶቹ ያላቸውን እውቀት፣አመለካከት እንቅስቃሴቸበተመለከተጥናት

በዳህረ ምረቃ ተማሪ በሆኑት አቶ ማዕከል ማይሌ በሚደረገው ጥናት መረጃ በማሰባሰብ ላይ እገኛሌሁ፡፡

ጥናቱ የሚካሄደዉ በቃልመጠይቅ ሲሆን የሚሰጡኝ መረጃዎች በሙሉ ምሰጥራዊታቸዉ በሚገባ የተጠበቀ ና ምላሽዎን የሚሰጡንፍቃደኛ እስከሆኑ ድረስ ብቻ ነው ፡፡ የሚሰጡን መረጃ በሚስጥር የሚያዝ መሆኑን አረጋግጥሌዎታለሁ፡፡ የእርስዎን ማንነት በሚስጥር ለመጠበቅ ሲባል ስምዎ በመጠይቅ ቅጹ ላይ አይጻፍም፡፡ እርስዎ በዚህ ጥናት ላይ በመሳተፍዎ የተነሳ ምንም ጉዳት እንደማይፈጠርብዎ እርግጠኛ ይሁኑ፡፡ በቃል መጠይቁ ላይ በከፊልም ይሁን ሙሉ በ ሙሉ አለመሳተፍ የሚችሉ ሲሆን ቃለ መጠይቁ የሚወስደው ጊዜ 15 ደቂቃዎችነው፡፡ በቃለ መጠይቁ ላይ ለመሳተፍ ፍቃደኛ ከሆኑ የሚሰጧቸው ምላሾች ለኢትዮጵያ የጤና ጥበቃ ሚኒስቴር እናየጤና አገሌግልት መስጫዎች ማሻሻያ የሚያግዙ ትክክለኛ ድምዳሜዎችና አስተያየቶች ላይ ለመዴረስ አስፈሊጊ በመሆኑ እባክዎ መመለስ ለሚፈሌጓቸው ጥያቄዎች ትክክለኛ ምላሾችዎን ይስጡን፡፡

የመጠይቁን ሙሉ ይዘቶች ተገንዝቤ በዚህ የምርምር ጥናት ፕሮጀክት ላይ ለመሳተፍ ፍቃደኛ ሆኛለሁ፡፡ አዎ….. አይደለም ------------------ ፍቃደኛ ስለሆኑ እናመሰግናለን

መመሪያ፡ከሚመርጡት መልስ እና ቀጥተኛ መልስ ከሚሰጡባቸው ጥያቄዎች ትይዩ የሚገኘውን ቁጥር ያክብቡ፣ መልስዎን በተሰጠው ቦታ ላይ ይጻፉ፡፡ክፍሌ1፡አርባምንጭ ሆስፒታል ውስጥ የቅድመወለድ ክትትል ከሚደረግላቸው ነፍሰጡር እናቶች መካከል ስለማህበራዊስና ህዝባዊ ባህሪያት በተመለከቱ ጥያቄዎች ምላሽ ሰጪዎች

| \| ተ/ቁ \| \| --- \| | ጥያቄ | አማራጭመልሶች |
| --- | --- | --- | --- |
| 101 | እዴሜዎ ስንት ነው? |  |
| 102 | የምን ሀይማኖት ተከታይ ነዎት? | ኦርቶድክስ -------1  ፕሮቴስታንት ----2  ካቶሉክ----------3  ሙስሊም--------4  ሌላካለ (ይግለጹ) |
| 103 | የጋብቻ ሁኔታዎ ምንድ ነው? | ያላገባች-----------1  ያገባች ------------2  የፈታች-------------3  የተለያየች-----------4  ባል የሞተባት -------5  አብራ የምትኖር-----6 |
| 104 | የትምህርት ደረጃዎ ምንድንነው? | ያልተማረች-----------1  ማንበብ ና መጻፍ የምትችል--2  አንደኛ ደረጃ ----------3  ሁለተኛ ደረጃ ያጠናቀቀች-----4  ኮሌጅ ወይም ዩኒቨርሲቲ ------5 |
| 105 | የትዳር አጋራዋ የትምህርት ደረጃዎ ምንድን ነው | ተማረ-----------1  ማንበብ ና መጻፍ የምችል-----2  አንደኛ ደረጃ ----------3  ሁለተኛ ደረጃ ያጠናቀቀ-----4  ኮሌጅ ወይም ዩኒቨርሲቲ -----5 |
| 106 | ስራዎ ምንድ ነው? | የመንግስት ሰራተኛ------------1  የቤት እመቤት -------------2  የግል ስራ ----------3  በግሌ ተቀጥሬ------4  ሌላ ካለ ይጥቀሱ_ |
| 107 | ወርሀዊ ገቢዎ ምን ያህል ብር ነው? | ---------ኢትዮ.ብር |

ክፍሌ 2፡የወሊድ ታሪክ መረጃ

| ተ/ቁ | ጥያቄ | አማራጭ መልሶች |
| --- | --- | --- |
| 201 | \| በጠቅላላው ምን ያህ ጊዜ አርግዘዋል ? \| \| --- \| | አንዴ -------------1  2-4----------------2  ከ4 በላይ ----------3 |
| 202 | በጠቅላላው ስንት ልጆች ወልደዋል | \| አልወለዴኩም --- 1  1------------------2  2 -----------------3 \| \| --- \|   ≥2----------------3 |
| 203 | ምን ያክል ልጆች በህይወት አሌዎት? | ምንም -------------1  1-2 ልጅ ------------2  2 እና ከዚያ በላይ ---3 |
| 204 | ወልድ ተጨናግፎቦዎት ያውቃል? | አዎ ----------------- 1  አያውቅም -----------2 |
| 205 | እርግዝናዎ ስንት ወር ይሆነዋል ? | < 4 ወራት ----------1  4-6 ወራት ----------2  7-9 ወራት ----------3 |

ክፍሌ3፡የቅዴመ ወለድ እንቅስቃሴዎች ግንዛቤ በሚመለከቱ ጥያቄዎች

| ተቁ | ጥያቄ | `አማራጭ መልሶች |  |
| --- | --- | --- | --- |
| 301 | ከመጸነስዎ በፊት የአካል እንቅስቃሴ ሰርተውያውቃሉ? | አዎ -------------1  አላውቅም ------2 |  |
| 302 | ስለቅድመ ወለድ የኣካል እንቅስቃሴዎች ሰምተውያውቃሉ? | አዎ -------------1  አላውቅም ------2 | አላውቅምከሆነወደቁ.304ይለፉ |
| 303 | ተቁ.302 መልስዎ አዎ ከሆነስለእንቅስቃሴዎቹከየትነውተማሩት (መረጃውያገኙት)? | ከጤናባለሙያ ------1  ከቤተሰብወይም ጓደኛ--2  ከመገናኛብዙሀን ----3  ከኢንተረኔት----------4  ከመጽሀፍ ----------5  ሌላካለይግለጹ |  |
| 304 | በተ.ቁ.302 መልስዎ አዎ ከሆነ ከሚከተለት ውስጥ የትኞቹን የቅዴመወሉዴ የአካል እንቅስቃሴዎች ያውቃል ወይም ሰምተዋል?  (ከአንዴ በላይ መልስ ሊሰጡ ይችላል) | መራመዴ/የእገርጉዞማዴረግሊ(Walk) -----1  ኤሮቢክስ-------------2  ማፍታታት/አየርመውሰድ------------------3  የዲላወሇሌእንቅስቃሴ------------------ 4  በጀርባ እንቅስቃሴ --5  የጉልበትናእግርእንቅስቃሴ-----6 |  |

ክፍሌ4፡የቅድመ ወሊድ እንቅስቃሴዎች ጠቀሜታ ያለ እውቀት በተመለከቱ ጥያቄዎች ምላሽ ሰጪዎች

| ተ/ቁ | ጥያቄ | አማራጭ መልሶች |
| --- | --- | --- |
| 401 | በእርግዝና ወቅት የአካል እንቅስቃሴ መስራት የጀርባ ህመምን ይቀንሳል፡፡  . | አዎ ------------1  አይደለም -----2  አላውቅም -----3 |
| 402 | በእርግዝና ወቅት የአካል እንቅስቃሴ መስራት ከመጠን በላይ የክብደት መጨመርን ይከላከላል፡፡ | አዎ -------------1  አይደለም -------2  አላውቅም ------3 |
| 403 | በእርግዝና ወቅት የአካልእንቅስቃሴመስራት ጉልበትና ብርታትን ይጨምራል፡፡ | አዎ ---------------1  አይደለም --------2  አላውቅም -------3 |
| 404 | የአካል እንቅስቃሴ መስራት በምጥና ወሊድወቅት የሚፈጠር ህመምን ይቀንሳል፡፡ | አዎ ---------------1  አይደለም ----------2  አላውቅም ---------3 |
| 405 | በእርግዝና ወቅትየአካል እንቅስቃሴ መስራት በእርግዝና የሚከሰት የስኳር በሽታን ይከላከላል/ይቀንሳል፡፡ | አዎ ---------------1  አይደለም ----------2  አላውቅም ---------3 |
| 406 | በእርግዝና ወቅት የአካል እንቅስቃሴ መስራት ከፍተኛ የደምግፊት እንዲይከሰተ ይካላከላል፡፡ | አዎ ---------------1  አይደለም ----------2  አላውቅም ---------3 |
| 407 | የአካል እንቅስቃሴ መስራት ከወሊድ በኋሊ ወዱያውኑ አቋምን በማስተካከል ያግዛል፡፡ | አዎ ---------------1  አይደለም ----------2  አላውቅም ---------3 |
| 408 | የአካል እንቅስቃሴ መስራት የቅዴመወሉዴና ድህረወሊድ ጭንቀትን ይከላከላል፡፡ | አዎ ---------------1  አይደለም ---------2  አላውቅም ---------3 |
| 409 | የአካል እንቅስቃሴ መስራት በጠቅላላ ጤንነትና ለህጻኑ እዴገት ጠቀሜታዎች አሉት፡፡ | አዎ ---------------1  አይደለም ---------2  አላውቅም ---------3 |
| 410 | በእርግዝና ወቅት ከማህፀን ደምመፍሰስ ሲያጋጥምዎ የአካል እንቅስቃሴ መስራት ኣይመከርም፡፡ | አዎ -----------------1  አይደለም ----------2  አላውቅም ---------3 |
| 411 | በእርግዝና ወቅት የምጥ ሕመም ወይም የሆድቁርጠት ሲኖር የአካል እንቅስቃሴ መስራት የተከለከነው፡፡ | አዎ ---------------1  አይደለም ----------2  አላውቅም ---------3 |
| 412 | የደረት ህመም ሲኖር በእርግዝናወቅት የአካልእንቅስቃሴ መስራት የተከለከለ ነው፡፡ | አዎ ---------------1  አይደለም ----------2  አላውቅም ---------3 |
| 413 | የመተንፈስ ችግር ሲኖር በእርግዝና ወቅት የአካል እንቅስቃሴ መስራት አይመከርም፡  . | አዎ ---------------1  አይደለም ----------2  አላውቅም ---------3 |
| 414 | ያለጊዜው ምጥ ሲፈጠር በእርግዝና ወቅት የአካል እንቅስቃሴ መስራት አያስፈልግም | አዎ ---------------1  አይደለም ----------2  አላውቅም ---------3 |
| 415 | በአግባቡ ቁጥጥር ያልተደረገበት የስኳር በሽታ ሲኖር በእርግዝና ወቅት የአካል እንቅስቃሴ መስራት የተከለከለ ነው፡፡ | አዎ ---------------1  አይደለም ----------2  አላውቅም ---------3 |
| 416 | በእርግዝና ወቅት ራስ ማዞር ሲኖር የአካል እንቅስቃሴ መስራት አይመከርም፡፡ | አዎ ---------------1  አይደለም ----------2  አላውቅም ---------3 |

ክፍሌ5፡በእርግዝናጊዜሰለሚደረጉ የአካል እንቅስቃሴ ስለተመለከቱ የአመለካከት ጥያቄዎች

| ተ/ቁ | ጥያቄ | በጣም እስማማለሁ | እስማማለሁ | አልወሰንኩም | አልስማማም | በጣም  አልስማማም |
| --- | --- | --- | --- | --- | --- | --- |
| 501 | በእርግዝና ወቅት የአካል እንቅስቃሴ መስራት አስፈሊጊ ነው ተብለው ይታምናል | 4 | 3 | 2 | 1 | 0 |
| 502 | በእርግዝና ወቅት አካላዊ እንቅስቃሴ ማዴረግ ህፃኑን ለአደጋ ያጋልጣ |  |  |  |  |  |
| 503 | የቅዴመወለድ የአካል እንቅስቃሴ ማዴረግ ከባህላችን ጋር ይስማማል |  |  |  |  |  |
| 504 | ነብሰጡር እናቶች በጤና ባለሙያዎች በመታገዝ የአካል ብቃት እንቅስቃሴ ማዴረግ አለባቸው |  |  |  |  |  |
| 505 | የቅዴመወሊዴ የአካል እንቅስቃሴ ማዴረግ ከእርግዝና ጋር የተዛመደ ችግሮችን ሊቀንስ ይችላል |  |  |  |  |  |
| 506 | በእርግዝና ወቅት የአካሌ ብቃት እንቅስቃሴ ማዴረግ ከድህረ ወሊድ በኋላ መልሶ ለማገገም ይረዳል |  |  |  |  |  |
| 507 | የቅዴመወለዴ እንቅስቃሴዎች ማዴረግ ወደ ቀደመው የሰውነት አቋም ወይም ቅርጽ እንደመሌስ ይረዳናል |  |  |  |  |  |
| 508 | በእርግዝና ወቅት የሚደረግ የአካል እንቅስቃሴ ስሌት ከአንዴ እናት ለላኛዋ እናት መለያየት አለበት |  |  |  |  |  |

ክፍሌ6፡ በነፍሰጡር እናቶች መሰራት ያለባቸው የአካል እንቅስቃሴዎችን የተመለከቱ ጥያቄዎች ምላሽ ሰጪዎች

| ተ/ቁ | ጥያቄ | አማራጭመልሶች |  |
| --- | --- | --- | --- |
| Q.601 | በአሁኑ እርግዝናዎ ወቅት የቅዴመወሊዴ የአካሌ እንቅስቃሴዎች ይሰራሉ? | አዎ ---------------1  አይደለም ----------2 | አይደለምወደ Q603 ይለፉ |
| Q.602 | አሁን እየሰሩ ያሉት የትኞቹን የአካል እንቅስቃሴ አይነቶች ነው?  (ከአንድ በላይ መልስመስጠት ይችላሉ) | የእገርጉዞማዴረግ(Walk) ------1  ኤሮቢክስ ----------------------2  መፍታታትና አየርመውሰዴ ----3  የዳሌ ወለል እንቀስቃሴ --------- 4  የጀርባ እንቅስቃሴ ---------------6  ጉልበትና የእግር ጣቶችን እንቀስቃሴ--7 |  |
| Q.603 | በአሁኑ እርግዛናዎ ወቅት የቅድመ ወለድየአካሌ እንቅስቃሴ እንዲሰሩ የመከርዎት (ያዘዙዎት) ማንነው? | ከጤናባለሞያ-------1  በራሴ --------------2  ለላ ሰው -----------3 |  |
| Q.504 | በሳምንትምንያህልጊዜየአካልእንቅስቃሴይሰራሉ? | <ሁለትጊዜ/ሳምንት---------1  > 3 ጊዜ/ሳምንት------2 |  |
| Q.605 | በአንድ የእንቅስቃሴክፍለ ጊዜ ለምን ያህል ደቂቃዎች የአካል እንቅስቃሴ ይሰራሉ? | < 20 ደቂቃ------------1  > 20---30 ደቂቃ-------2  ከ30 ደቂቃ በላይ |  |
| Q.606 | በአሁኑ እርግዝናዎ ወቅት የአካል እንቅስቃሴ የማይሰሩበት ምክንያት ምንድንነው? (ከአንድበላይመልስመክበብይችላል) | 1.የጤና ባለሙያዬ የአካሌ እንቅስቃሴ እንድሰራ አልመከረኝም  2. ምንምመረጃ/ግንዛቤየለኝም  3.የአካል እንቅስቃሴ መስራት ለህጻኑ ጤና ጎጂነው  4. የጊዜ እጥረት  5.የአካል እንቅስቃሴ መስራት ለእኔ ጉዳት አለው  6. የአካል እንቅስቃሴ መስራት ይደክመኛል  7. በጥሩ የጤና ሁኔታ ላይ አልገኝም |  |

ስለተሳትፎዎ አመሰግናለሁ**!!!**
