# Supplementary material for: Factors associated with antenatal exercise in Arba Minch town, Southern Ethiopia: A community-based cross-sectional study
Source: PLoS One. 2022 Feb 22;17(2):e0260840. doi: 10.1371/journal.pone.0260840 (PMC8863279; doi:10.1371/journal.pone.0260840)
Supplement: Attachment — Submitted filename: The rebuttal letter to PLOSE ONE.docx [file pone.0260840.s003.docx]

**September 18, 2021**

Rebuttal letter for paper [PONE-D-21-11918] - [EMID:153efb5d1967c1c4] “**Practice of antenatal exercise and its associated factors among pregnant women in Arba Minch town, Southern Ethiopia: A community based cross-sectional study**“

**Dear Reviewers,**

First, we want to thank you for giving your time and attention for our paper. Each and every comment given by academic editor and reviewers helped us to understand how we can improve our paper. We have modified and revised the manuscript according to the comments of reviewers. The changes made were highlighted on the revised manuscript with a track changes. Below, we want to respond for each raised comments. The following table is split in to the categories to achieve a better structure for our responses for each comments of academic editor, reviewer 1, and reviewer 2. This changes have improved the manuscript considerably, and we hope that it can be published without delay

| For academic editor | |
| --- | --- |
| Raised requirements. | Responses |
| 1. Please ensure that your manuscript meets PLOS ONE's style requirements, including those for file naming. | Authors corrected the manuscript based on the PLOS ONE style templets. We highlighted the corrected. |
| 2. Please provide additional details regarding participant consent. In the ethics statement in the Methods and online submission information, please ensure that you have specified what type you obtained (for instance, written or verbal, and if verbal, how it was documented and witnessed). If your study included minors, state whether you obtained consent from parents or guardians. If the need for consent was waived by the ethics committee, please include this information. | Authors corrected that the consent obtained was verbal because some of respondents cannot read and write and their consent was recorded with questionnaire. Regarding the minors in age , the minimum age in this study was 16 (3 individuals) and 17(11 individuals). For minors, the consent was taken from parents. |
| 3. We note that you have indicated that data from this study are available upon request. PLOS only allows data to be available upon request if there are legal or ethical restrictions on sharing data publicly. For more information on unacceptable data access restrictions, please see <http://journals.plos.org/plosone/s/data-availability#loc-unacceptable-data-access-restrictions>.   In your revised cover letter, please address the following prompts:  a) If there are ethical or legal restrictions on sharing a de-identified data set, please explain them in detail (e.g., data contain potentially sensitive information, data are owned by a third-party organization, etc.) and who has imposed them (e.g., an ethics committee). Please also provide contact information for a data access committee, ethics committee, or other institutional body to which data requests may be sent.  b) If there are no restrictions, please upload the minimal anonymized data set necessary to replicate your study findings as either Supporting Information files or to a stable, public repository and provide us with the relevant URLs, DOIs, or accession numbers. For a list of acceptable repositories, please see <http://journals.plos.org/plosone/s/data-availability#loc-recommended-repositories>.  We will update your Data Availability statement on your behalf to reflect the information you provide. | There are no ethical or legal restrictions on sharing a de-identified data set, therefore we upload the anonymized SPSS data set as Supporting Information files. |
| 4. Please include a caption for figure 1. | A caption for figure 1 is include. |
| 5. Please include your tables as part of your main manuscript and remove the individual files. Please note that supplementary tables (should remain/ be uploaded) as separate "supporting information" files | All tables are included as the part of your main manuscript and the individual files were removed. |
| 6. Please review your reference list to ensure that it is complete and correct. If you have cited papers that have been retracted, please include the rationale for doing so in the manuscript text, or remove these references and replace them with relevant current references. Any changes to the reference list should be mentioned in the rebuttal letter that accompanies your revised manuscript. If you need to cite a retracted article, indicate the article’s retracted status in the References list and also include a citation and full reference for the retraction notice. | All reference list were reviewed and retracted references were removed (eg. Reference 3 and 7) and replaced by the current references (ref. 3, 7 and 20). |
| For reviewers | |
| Reviewer 2   1. Title – very long - Practice of antenatal exercise and its among pregnant women in Arba Minch town, Southern Ethiopia: A community based cross-sectional study Suggestion - Factors associated with antenatal exercise in Arba Minch town, Southern Ethiopia: A community based cross-sectional study | Title was modified as suggested “Factors associated with antenatal exercise in Arba Minch town, Southern Ethiopia: A community based cross-sectional study |
| 2. Abstract - I suggest that the results with positive or negative association according to the PE practice be presented separately, both in the results and in the conclusion Ethics – “informed consent was taken” - Written or verbal? If verbal, how was this obtained and recorded? Introduction | Results with positive or negative associations were presented separately in abstract, result and conclusion sections and regarding ethics, it was corrected as a verbal consent and recorded in the questionnaire as it explained in the above comment by editors. |
| 3. Introduction – please update your data Reference 3  Reference 7 -. | Data were updated according to the given current references. |
| 4. Method: Sample size – “adequate practice of 95 antenatal exercise to be 50%” - Very high 50%, what is the reference used?   I also suggest that you include a figure that explains the representativeness of the sample in different districts, sub-districts, this was not made explicit and can be very well explained with a figure or even a map | At the time of the study, authors lack the proportion of antenatal exercise for sample size calculation, so that we used 50% to get large sample size . |
| 5. Results – Will the tables not be shown in the results? They are all in the supporting information. I believe it is fundamental to see the numbers of the data presented and the tables must appear in the text, especially 5, 6 and 7 with the analyses | Authors inserted all tables in the main manuscript as it is also raised by the academic editor. |
| 6. And it needs to be very clear which associations are positive and which are negative (this in the results, discussion and conclusion) | We discussed positively and negatively associated factors separately. |
| 7. Discussion It also needs to contextualize more in the discussion the importance of marriage, education of the woman and the husband in the society where the study was carried out.   And in the limitations, further reinforce the question of the representativeness of the sample, that is, how much it represents the city, region or country where the study was carried out. | The association of educational status of husbands’ on the practice of ANEx is discussed and contextualized in the study setting. But, the marriage status and educational status of women did not have statistical association with ANEx.  This study only assessed the practice of ANEx to the pregnant women in the town area, therefore it can represent the population in town.  This issue is discussed in the limitation section. |

Sincerely,

MAECHEL MAILE BEYENE

Department of Midwifery,

College of Medicine and Health Sciences,

Arba Minch University, Arba Minch, Ethiopia.

Email: [maechelmaile11@gmail.com](mailto:maechelmaile11@gmail.com)
